# Atypical Mycobacterium fortuitum Ulcer in an Immunosuppressed Patient With Lupus: A Case Report

**DOI:** 10.7759/cureus.106807

**Published:** 2026-04-10

**Authors:** Allison C Eaton, Raj H Patel, Maria I Longo

**Affiliations:** 1 Medicine, Nova Southeastern University Dr. Kiran C. Patel College of Osteopathic Medicine, Davie, USA; 2 Dermatology, HCA Healthcare/USF Morsani College of Medicine GME, Largo, USA; 3 Dermatology, University of Florida College of Medicine, Gainesville, USA

**Keywords:** atypical mycobacterial skin infection, cutaneous bacterial infection, cutaneous nontuberculous mycobacterial infection, delayed wound healing, diagnostic and therapeutic challenge, mycobacterium fortuitum, systemic lupus erythematosus

## Abstract

A 65-year-old woman with systemic lupus erythematosus (SLE) on methotrexate and prednisone presented to the dermatology clinic with a chronic, painful ulcer on the right lower leg. The initial differential diagnosis included pyoderma gangrenosum and vasculitis. However, biopsy and wound cultures ultimately revealed *Mycobacterium fortuitum*. The patient was treated successfully with moxifloxacin and trimethoprim-sulfamethoxazole for four months, resulting in complete ulcer resolution. This case highlights the importance of considering atypical mycobacterial infection in chronic non-healing ulcers, particularly in immunosuppressed patients, and demonstrates the need for early tissue cultures to guide appropriate antimicrobial therapy.

## Introduction

Systemic lupus erythematosus (SLE) is a chronic autoimmune disease characterized by immune-mediated multisystem involvement, including mucocutaneous, vascular, musculoskeletal, and renal manifestations [1]. Management often requires long-term immunomodulatory or immunosuppressive therapy, including hydroxychloroquine and additional agents for more severe disease [1]. Although these therapies reduce disease activity and prevent organ damage, they increase susceptibility to opportunistic infections.

Chronic lower extremity ulcers are a common clinical problem and may result from venous insufficiency, arterial disease, inflammatory conditions, trauma, or infection [2]. In patients with systemic disease, ulcer formation is often multifactorial and complicated by impaired wound healing. Treatment is typically directed toward the presumed etiology, such as compression for venous disease, immunosuppression for inflammatory ulcers, or antimicrobial therapy when infection is suspected [2]. Failure to respond to standard management should prompt reconsideration of the diagnosis and evaluation for atypical infectious causes.

Non-tuberculous mycobacteria (NTM) are environmental organisms that have emerged as increasingly important pathogens in dermatologic practice. The incidence of cutaneous NTM infections in the United States has risen substantially in recent decades, with rates estimated at 0.9-2.0 cases per 100,000 persons and a nearly threefold increase since the 1980s, as reported in a recent review [3]. Regional variability has also been observed, with the Gulf Coast identified as having the highest reported prevalence of extrapulmonary NTM infections in the country [4]. These infections can present with diverse and often nonspecific clinical and histopathological features, including papules, nodules, plaques, abscesses, and ulcers, often mimicking bacterial, fungal, or inflammatory dermatoses, thereby complicating timely diagnosis [3-5].

Immunosuppressed individuals, including those receiving corticosteroids or immunomodulatory therapies, are particularly susceptible to cutaneous manifestations and atypical disease courses [3]. In this report, we describe a case of *Mycobacterium fortuitum* ulceration in a patient with SLE, initially misattributed to autoimmune disease, which was ultimately resolved with culture-directed antimicrobial therapy.

## Case presentation

A 65-year-old woman with a history of SLE presented to the dermatology clinic with an enlarging, tender ulcer on the right lateral leg. She denied any history of trauma to the site. She also reported the recent development of two other ulcers (one on the left foot and the other on the right ankle), which had spontaneously healed. Two weeks prior to this visit, her rheumatologist had switched her from oral prednisone to an oral prednisone taper, but there was no improvement in the size of the ulceration. Her lupus was otherwise well controlled on methotrexate and prednisone, and she had no prior history of cutaneous lupus.

Given her history of autoimmune disease and immunosuppressive therapy, the initial differential diagnosis favored inflammatory or vasculopathic ulceration related to SLE. The absence of fever, systemic symptoms, or antecedent trauma further supported a noninfectious working diagnosis at presentation.

On physical examination, she was afebrile. Skin findings revealed a 6 × 2 cm erythematous, ulcerated plaque on the right lateral leg with some granulation tissue present at the base, surrounded by a prominent rim of erythema. Laboratory results were unremarkable, and a recent arterial Doppler showed no evidence of arterial occlusive disease, excluding significant peripheral arterial disease as an underlying cause.

A punch biopsy of the wound edge showed superficial and deep perivascular lymphoplasmacytic infiltrate with focal neutrophils. The biopsy was performed to differentiate between inflammatory, infectious, and malignant etiologies of a nonhealing ulcer. Periodic acid-Schiff (PAS) and Grocott-Gomori Methenamine Silver (GMS) stains were negative, making fungal infection less likely. Initial treatment included topical corticosteroids, and the patient continued her immunosuppressive regimen, as the histology findings were interpreted as most consistent with an inflammatory process.

**Figure 1:**
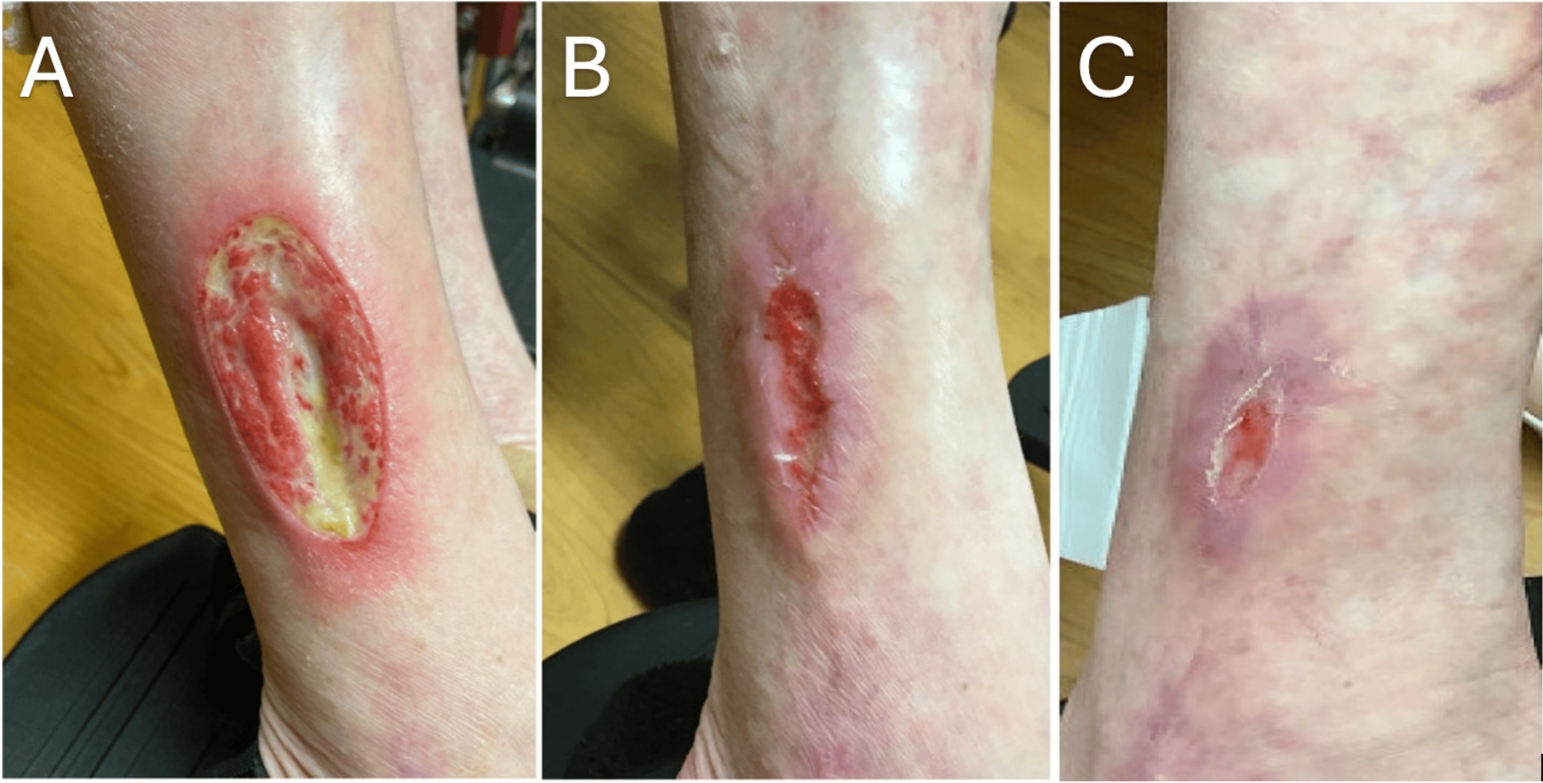
(A) Initial presentation of a painful, well-demarcated ulcer on the right lower leg with a fibrinous yellow base, surrounding erythema, and inflammatory rim, (B) three-month progression of healing after starting oral antibiotic therapy showing wound edge contraction, decreased surrounding inflammation, and reduction in ulcer size (C) six-month stage showing near-complete re-epithelialization with residual post-inflammatory erythema and scar formation.

Due to lesion progression despite therapy, surgical debridement was performed, and tissue was sent for culture, as treatment resistance raised concern for an occult infectious etiology. After 10 days, mycobacterial culture grew *Mycobacterium fortuitum*. Isolation of a rapidly growing nontuberculous mycobacterium provided an explanation for the lesion’s chronicity and lack of response to immunosuppressive treatment. Given the heterogeneous resistance profiles characteristic of nontuberculous mycobacteria, antimicrobial susceptibility testing was performed to inform targeted treatment. Results indicated that the organism had sensitivity to amikacin, ciprofloxacin, linezolid, moxifloxacin, and trimethoprim-sulfamethoxazole. Therapy was subsequently initiated with oral moxifloxacin and trimethoprim-sulfamethoxazole in accordance with the susceptibility results. Her course of oral prednisone was tapered. The ulcer resolved following a four-month course of antibiotic therapy and non-adherent bandaging with petrolatum (Figure 1).

## Discussion

Chronic leg ulcers are a source of substantial morbidity and can significantly impair quality of life. The differential diagnosis for these ulcers is broad and includes vascular disorders, neuropathies, autoimmune or inflammatory conditions, and infectious diseases [6]. In cases where routine aerobic and anaerobic cultures are sterile, and patients fail to respond to antibiotics for pyogenic infection, the possibility of mycobacterial infection should be considered [7].

NTM, which include *Mycobacterium fortuitum*, *Mycobacterium chelonae*, and *Mycobacterium abscessus*, are the cause of various skin, soft tissue, and bone infections [8-10]. *Mycobacterium fortuitum* is a rapidly growing environmental mycobacterium, ubiquitously present in soil and water [11]. These organisms commonly manifest as localized abscesses or chronic ulcers and may mimic inflammatory dermatoses clinically. Disseminated disease may occur in immunocompromised patients [12]. Infection typically begins following trauma to the skin, whether accidental or surgical [7,12]. *Mycobacterium fortuitum* infections have been described following various procedures such as dermatologic surgery, pedicures, intravenous catheter use, breast implantation, acupuncture, and subcutaneous injections [12].

Histological findings are varied and nonspecific; thus, microbiological confirmation with wound cultures is paramount to definitive diagnosis [5]. The identification of this agent requires a Ziehl-Neelsen stain for acid-fast bacilli and culture on Lowenstein-Jensen media, as routine cultures may fail to detect this mycobacterial organism [7]. Tissue cultures may take weeks to yield results; acid-fast staining is commonly used to aid diagnosis [5]. In the present case, progression despite immunosuppressive therapy prompted further evaluation and ultimately led to the identification of *Mycobacterium fortuitum* on mycobacterial culture. 

Optimal treatment of NTM is not well established, and specific guidelines are not available [12,13]. Antibiotic management is species-dependent and guided by susceptibility results, generally requiring a multi-drug regimen for several months with potential adjunctive surgical debridement [12,13]. To prevent relapse, a combination of two agents for at least four months is typically recommended for *Mycobacterium fortuitum* infection. *Mycobacterium fortuitum* has frequently demonstrated sensitivity to agents such as amikacin, tetracyclines, first-generation cephalosporins, quinolones, and newer macrolides [5,14]. In this patient, targeted therapy with moxifloxacin and trimethoprim-sulfamethoxazole resulted in complete clinical resolution.

The prevalence of non-tuberculous mycobacterial infections is increasing and likely underestimated, given the varied clinical presentations consisting of both cutaneous and pulmonary disease [3,5]. While these infections are more common in immunocompromised individuals, they can also affect immunocompetent patients. Given their association with common surgical procedures and accidental trauma, and their increased pathogenicity in immunocompromised hosts, heightened clinical suspicion is essential. Due to the lack of routine testing for mycobacterial cultures on skin biopsy specimens or wound cultures, diagnosis is frequently delayed [4,12]. Delayed recognition may contribute to prolonged disease course and inappropriate initial management. Microbiological identification with susceptibility testing facilitates the selection of targeted antimicrobial therapy. Further studies are needed to compare the efficacy of different antibiotic regimens and to establish evidence-based treatment strategies aimed at reducing morbidity associated with these infections.

## Conclusions

This case highlights the importance of considering NTM infections in the differential diagnosis of chronic, non-healing ulcers, particularly in immunosuppressed individuals. Due to their nonspecific clinical presentation and frequent misdiagnosis, NTM infections require a high index of suspicion and early microbiologic evaluation. Prompt tissue culture and susceptibility testing can guide effective multidrug therapy, as demonstrated by the complete resolution of *Mycobacterium fortuitum* ulceration in this patient.
